# Development and validation of immunoassay for whole cell detection of *Brucella abortus* and *Brucella melitensis*

**DOI:** 10.1038/s41598-020-65347-9

**Published:** 2020-05-22

**Authors:** Richa Hans, Pranjal Kumar Yadav, Pushpendra Kumar Sharma, Mannan Boopathi, Duraipandian Thavaselvam

**Affiliations:** Defence Research and Development Establishment, DRDO, Jhansi Road, Gwalior, 474002 India

**Keywords:** Kinetics, Bacterial infection

## Abstract

*Brucella* is alpha-2 Proteobacteria mainly responsible for multi-factorial bacterial zoonotic disease brucellosis with low concentration (10–100 CFU) required to establish the infection. In this study, we developed sandwich ELISA with detection range of 10^2^ to 10^8^ cells mL^−1^ and limit of detection at 10^3^ cells mL^−1^ by employing polyclonal rabbit IgG (capture antibody, 10 µg mL^−1^) and mice IgG (detection antibody, 50 µg mL^−1^) antibody for its detection. Surface Plasmon Resonance evaluated the interaction of detection antibody with whole cell spiked serum samples at LOD of 10^2^ cells mL^−1^ along with non co-operative interaction of protein albumin. Further, kinetic evaluation study using detection antibody against cell envelope antigen was performed whereby, Equilibrium Dissociation Constant (K_D_) and Maximum Binding Capacity (B_max_) were found to be 16.48 pM and 81.67 m° for *Brucella abortus* S99 and 0.42 pM and 54.50 m° for *Brucella melitensis* 16 M, respectively. During interference study, sandwich ELISA assay cross-reacted with either of the polyclonal antibody of above *Brucella* species. Upon validation, no cross-reactivity observed with bacteria-closely related to *Brucella*. In conclusion, developed semi-quantitative sandwich immunoassay is sensitively rapid in whole cell detection of *Brucella* and will be useful in development of detection assays from environmental and clinical matrices.

## Introduction

Brucellosis is re-emerging infectious bacterial zoonosis caused by small gram-negative, cocco-bacilli, facultative intracellular bacteria belongs to genus *Brucella*^1^. It is 0.5–0.7 µm in diameter and 0.6–1.5 µm in length, first reported by Sir David Bruce from military personnel in Malta^2,3^. It causes contagious reproductive disease in livestock and predominantly transmitted to human through *Brucella melitensis*, *Brucella abortus*, *Brucella suis* and *Brucella canis*^4,5^. *Brucella* outer membrane proteins (OMPs) major virulent determinants facilitate infection and elicits cell-mediated and humoral response enabling early recognition in acute cases^6,7^. Its transmission perpetuates through direct contact and by consuming contaminated raw or under-cooked animal products^8^. Disease eradication delays due to its poor sero-prevalence and being un-reported and under-diagnosed^9,10^. Diagnostic threshold relies on combinatorial serological tests including Rose Bengal Test (RBT), Serum Agglutination Test (SAT) and Complement Fixation Test (CFT), although sensitive but suffers high false-positive rates in chronic cases^11^. Biochemical and Bacteriophage Typing tests are gold standard to detect *Brucella* as type-biovars^12^. PCR based molecular approach as URS-PCR with unique repeat sequence locus on chromosome 1 of *Brucella* specifically differentiate *Brucella abortus* and *Brucella melitensis* at species (spp.) level^13^. For the detection of *Brucella* in water and buffalo milk samples, standard serological and microbiological methods were compared with molecular assays to evaluate the feasibility of PCR and rtPCR methods in combined use rather than one test alone for detection^14^. Also, techniques like Lateral Flow assay, Multiplex and quantitative Real Time PCR (rtPCR, qPCR) and Isothermal based amplification are rapidly used along with gold standard for reporting incidence of low-disease burden in pre-clinical and sub-cellular intracellular infections^15–17^. The molecular based detection usually requires person-in-expertise for handling and sample pre-treatments made it difficult for employing in field applications^18^. However, a novel isothermal amplification technique of multiple cross displacement amplification (MCDA) coupled with nanoparticles-based lateral flow biosensor (LFB) potentially resulted in specific detection of *Brucella* (targeting *Bscp31* species-specific gene) with simple and rapid visual detection of *Brucella*-specific amplicons^19^. The serological tests and true gold standard alone cannot be defined as appropriate to differentiate *Brucella* related or non-related infections with accuracy due to low specificity in endemic areas^20^. Although the sensitivity of indirect and quantitative ELISA is well-known in standard determination of anti-LPS antibody, recently a new indirect ELISA was reported with high diagnostic performance of sensitivity (PPV = 95.7%) and specificity (NPV = 97.8%) based on whole cell (WC) *Brucella abortus* S99 lysate for IgM anti-*Brucella* antibody detection in human serum^21^. A new colorimetric immunoassay based on colored nanoparticles conjugated with polyclonal antibody against *Brucella abortus* was also developed to detect *Brucella* WC antigen with detection range of 1.5 ×10^3^ to 1.5 ×10^8^ CFU mL^−1^ at limit of detection (LOD) of 450 CFU mL^−1^^22^. The polyclonal antibody (pAb) as a capture antibody offers optimal maximum trap rate and intensify capture opportunities for more sensitivity of the assay^23^. Sandwich ELISA (S-ELISA) measures protein, antibodies (Ab) and cytokines (IFN-gamma) as a serial or parallel test for disease diagnostics in early detection^24,25^. In addition, chemically modified immuno-sensors based on conventional ELISA detecting IgG1 Ab (characteristic of acute and chronic stage) to elucidate the health related complexities in a timely manner were reported^26–28^. Bio-sensors are simple, accurate with high speed, specific and sensitive in detection to provide direct sample testing with considerable analytical interests^29^. Surface Plasmon Resonance (SPR) validates immobilization of IgG antibody, viable WC and DNA by detecting high molecular analytes whereby ligand binding (association) increases mass on the chip surface and on dissociation decreases the mass. Such changes produce direct detectable signal by affecting the refractive index, RI^30,31^. *Escherichia coli* detection by SPR was reported by immobilizing anti-*Escherichia coli* antibody having LOD at 10^4^ CFU mL^−1^ with functionalized gold substrate (acid-thiol) and 10^3^ CFU mL^−1^ with gold nanoparticles respectively^32^. Recently, SPR detected immune responses in dengue virus (DENV) infection and modified (SiO_2_-coated) SPR chip detected WC with sensitive limit of 6 ×10^−7^ refractive index unit, RIU^33–35^. Since, cell surface proteins are most appropriate target in pathogen detection. Thus, in our proposed study, we have raised pAbs against intact whole cell of *Brucella* and developed a semi-quantitative S-ELISA employing SPR based evaluation and validation (with WC spiked serum) of mice IgG detection antibody in direct whole cell detection of *Brucella*. Also, we characterized these high affinity antibodies in recognizing certain cell-surface components to develop a conventional S-ELISA for fast, direct and convenient detection of *Brucella*.

## Results

### **Production of pAbs against*Brucella*WC Ag**

The obtained Ab titer (see Supplementary Fig. S1a to d online) with whole cell based I-ELISA for mice and rabbit is ≥ 64000 with OD value comparatively more with anti-sera raised against WC Ag of *Brucella abortus* S99. Titer of Ab defines to be the reciprocal of the highest Ab dilution that presents an absorbance greater than or equal to 2.1 fold of the background absorbance (i.e. blank OD value or negative control)^23^. Polynomial regression co-efficient value (R^2^) obtained is almost equal to 1 with linear model equation, y = − 0.009×^4^ + 0.160×^3^ − 0.878×^2^ + 2.037×− 1.189 with R^2^ = 0.999 and y = − 0.019×^3^ + 0.233×^2^ − 0.181× + 0.103 with R^2^ = 0.994 for mice and rabbit pAb titer raised against *Brucella abortus* S99 and y = 0.012×^3^ − 0.085×^2^ + 0.252×− 0.130 with R^2^ = 0.999 and y = − 0.005×^3^ + 0.066×^2^ + 0.071× + 0.266 with R^2^ = 0.995 respectively against *Brucella melitensis* 16 M having linearity with best fit for capturing data pattern at low bias.

### SDS-PAGE and western blot analysis of purified IgG pAbs

Purified pAbs (WC Ag raised pAbs) developed against both *Brucella* spp. at 10 µg of protein concentration were applied on 12% SDS gel and analysed as two fragments of IgG Ab (see Supplementary Fig. S2a online) of heavy (50 kDa) and light (25 kDa) chains^36^. IPA-TCA precipitation resulted in the removal of albumin protein (72 kDa) and purified IgG pAb was obtained (see Supplementary Fig. S2b online). Cell envelope (CE) and whole cell sonicated (SA) Ag at 5 µg protein concentration were applied on 12% SDS gel showing all groups 1, 2 and 3 major proteins of *Brucella* (see Supplementary Fig. S2c,d online). Characterization with western blot revealed that group 1 were the prominent proteins against which mice IgG detection pAb was reacting at 1:100 dilution in both the *Brucella* spp. respectively (see Supplementary Fig. S2e,f online).

### WC based S-ELISA for *Brucella* detection

The OD values at 495 nm were analysed with standard curve of polynomial regression co-efficient value R^2^ = 1, best fit for above set of pAbs having comparable and selective response with linear association at low bias having linear model equation, y = 0.033×^3^ − 0.306×^2^ + 1.045×− 0.615 with R^2^ = 1 and, y = 0.002×^5^ − 0.060×^4^ + 0.564×^3^ − 2.494×^2^ + 5.386×− 3.290 with R^2^ = 0.996 in WC S-ELISA (Supplementary Fig. S3) for detection of *Brucella abortus* S99 and *Brucella melitensis* 16 M at 2 fold concentrations of mice IgG detection Ab respectively. In developed S-ELISA (see Supplementary Fig. S3a,b online), rabbit IgG pAb at 10 µg mL^−1^ with 2 fold serial dilution of mice IgG pAb at 100 µg mL^−1^ is effective in detecting 1.2 ×10^8^ CFU mL^−1^ WC Ag of both *Brucella* spp. Almost similar detection was obtained with 100 µg mL^−1^ and 200 µg mL^−1^ concentration of mice IgG pAb (see Supplementary Fig. S3b online) on comparing the OD values of optimized S-ELISA. Therefore, rabbit IgG pAb at concentration 10 µg mL^−1^ and mice IgG pAb at 100 µg mL^−1^ were sensitive in detecting WC of *Brucella* spp.

### Checker-board S-ELISA for determining detection range and LOD

**Figure 1:**
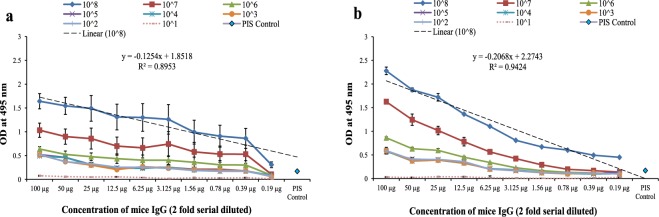
Checker-board S-ELISA for determining detection range and LOD for two *Brucella* spp. **(a,b)** Standard curve showing y-axis (absorbance at 495 nm) and x-axis showing 2 fold serial diluted concentrations of mice IgG detection Ab (100 µg mL^−1^ to 0.19 µg mL^−1^) detecting 10 fold serial diluted WC Ag of *Brucella* spp. (10^8^ CFU mL^−1^ to 10^1^ CFU mL^−1^) along the checker-board combinations. Linear regression co-efficient, R^2^ = 0.8953 with linear model equation, y = − 0.1254×+1.8518 and R^2^ = 0.9424 with model equation, y = − 0.2068×+ 2.2743 for both *Brucella abortus* S99 and *Brucella melitensis* 16 M respectively. Mice pre-immune sera (PIS at dilution 1:1000) as an experimental control was used in the study.

Standard checker-board S-ELISA was performed with different concentration of *Brucella abortus* S99 and *Brucella melitensis* 16 M inactivated WC Ag, detected by 2 fold serial dilution of detection Ab (Fig. 1a,b). WC were detected with different concentration of mice IgG detection Ab to obtain a standard curve best fit to linear model equation y = − 0.1254×+ 1.8518 for *B. abortus* S99 and y = − 0.2068×+ 2.2743 for *B. melitensis* 16 M respectively. Standard curve with linear regression co-efficient of R^2^ = 0.8953 and R² = 0.9424 respectively, is the working range (i.e. Detection range, 10^2^ µg mL^−1^ to 1.56 µg mL^−1^ at detection limit of 10^3^ CFU mL^−1^ for *B. abortus* S99 and 10^2^ µg mL^−1^ to 0.78 µg mL^−1^ at detection limit of 10^3^ CFU mL^−1^ for *B. melitensis* 16 M) of the standard assay. LOD is derived as; Ab_LOD_ = Mean Ab_blank_ + 3(σ_blank_), where Ab_LOD_ is the optical density (OD) corresponds to LOD, OD of blank is Ab_blank_ and, calculated standard deviation from repeated assay data (in duplicates) corresponds to σ_blank_ as; Ab_LOD_ = 0.042 + 2(0.024) i.e. 0.090 for *Brucella abortus* S99 and OD Ab_LOD_ = 0.044 + 2(0.024) i.e. 0.092 for *Brucella melitensis* 16 M (corresponding Ab_LOD_ at 10^3^ CFU mL^−1^)^23^.

### Interference study for cross-reactivity and evaluation of effective concentration of detection and capture antibody

**Figure 2:**
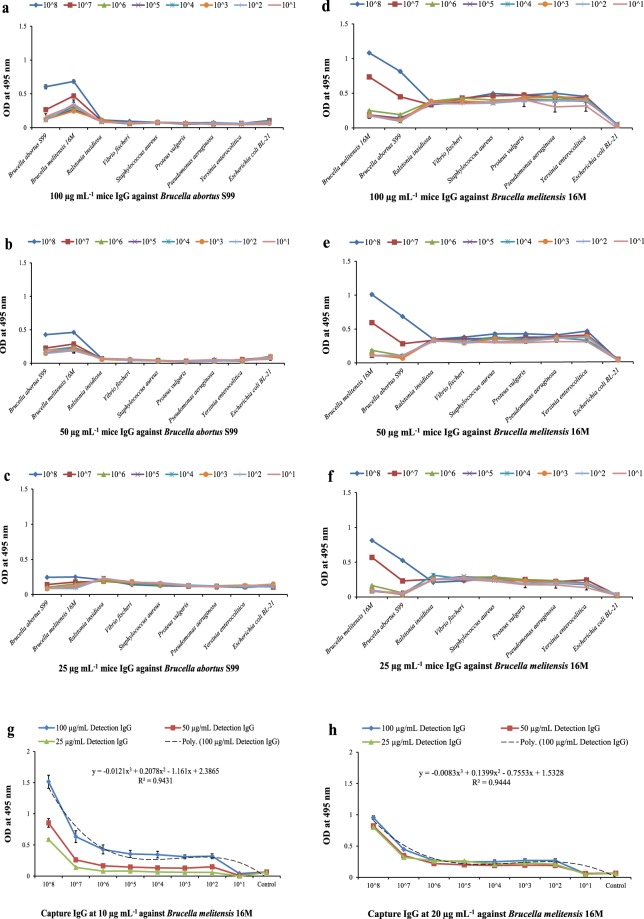
Interference study for determining cross-reactivity related to *Brucella* spp. with different concentration of WC mice IgG detection Ab and evaluation of effective working concentration of rabbit IgG capture Ab using developed S-ELISA. **(a,b,c)** Seven closely *Brucella* related bacterial spp. along with *Brucella melitensis* 16 M for S-ELISA against *Brucella abortus* S99 at 100, 50 and 25 µg mL^−1^ of mice IgG detection Ab along x-axis. **(d,e,f)** Seven closely *Brucella* related bacterial spp. along with *Brucella abortus* S99 for S-ELISA against *Brucella melitensis* 16 M at 100, 50 and 25 µg mL^−1^ of mice IgG detection Ab along x-axis. The y-axis showing absorbance at 495 nm respectively. **(g,h)** The WC rabbit IgG capture Ab at 10 and 20 µg mL^−1^ concentration with 50 µg mL^−1^ of mice IgG detection Ab against *Brucella melitensis* 16 M detecting 10 fold serial diluted (10^8^ CFU mL^−1^ to 10^1^ CFU mL^−1^) WC Ag of *Brucella melitensis* 16 M along x-axis respectively. The y-axis showing absorbance at 495 nm. Polynomial regression co-efficient value for capture Ab at 10 µg mL^−1^ is R^2^ = 0.9431 with model equation, y = − 0.0121×^3^ + 0.2078×^2^ − 1.161×+ 2.3865 and for capture Ab at 20 µg mL^−1^ concentration is R^2^ = 0.9444 with model equation, y = − 0.0083×^3^ + 0.1399×^2^ − 0.7553×+ 1.5328 for *Brucella* WC detection.

*Brucella abortus* S99 and *Brucella melitensis* 16 M along with bacteria closely related to *Brucella* spp. (Table 1) were evaluated for cross-reactivity and for determining the effective (best working) concentration of mice IgG detection Ab and rabbit IgG capture Ab with minimal cross-reactivity for the developed assay (Fig. 2a–f). On measuring the absorbance values, effective concentration was found to be 50 µg mL^−1^ for mice IgG detection Ab. Concentration less than 50 µg mL^−1^ (≤ 0.2 OD value in S-ELISA detecting *Brucella abortus*) and more than 50 µg mL^−1^ (≥ 0.5 OD value in S-ELISA detecting *Brucella melitensis*) were cross-reactive. And, minimum cross-reactivity was observed with bacteria closely related to *Brucella* spp. at 50 µg mL^−1^ of detection Ab. On evaluating capture antibody at 10 and 20 µg mL^−1^ of concentration with 50 µg mL^−1^ of optimized detection antibody (Fig. 2g,h), OD at 495 nm was analysed with standard curve of polynomial regression co-efficient best fit to linear equation, y = − 0.0121×^3^ + 0.2078×^2^ − 1.161×+ 2.3865 with R^2^ = 0.9431 and, y = − 0.0083×^3^ + 0.1399×^2^ − 0.7553×+ 1.5328 with R^2^ = 0.9444 for the two concentrations respectively. On comparing the OD values experimentally, it was found that 10 µg mL^−1^ of capture antibody sensitively detected WC Ag of *Brucella* at detection range of 10^2^ to 10^8^ CFU mL^−1^ with LOD at 10^3^ CFU mL^−1^ respectively. The pAbs raised against WC of two *Brucella* spp. were cross-reacting in detection of either of the *Brucella abortus* and *Brucella melitensis* sensitively within the developed assay. Therefore, S-ELISA developed with rabbit IgG capture Ab at 10 µg mL^−1^ and mice IgG detection Ab at 50 µg mL^−1^ at LOD of 10^3^ CFU mL^−1^ was cross reacting with both *Brucella abortus* and *Brucella melitensis* at spp. level.

**Table 1:** The list of *Brucella* spp. and closely *Brucella* related bacterial spp. used in the study with their CFU count mL^−1^ at different dilutions respectively.

Sr. No.	Bacterial Species/Strain	ATCC/MTCC/NCTC Call No.	(CFU mL^−1^) Counted	Counting Dilutions
1.	*Brucella abortus* (S99 Strain)	NCTC 11363	3.1 ×10^9^ CFU mL^−1^	10^−6^
2.	*Brucella melitensis* (16 M Strain)	NCTC 10094	1.8 ×10^9^ CFU mL^−1^	10^−6^
3.	*Escherichia coli* (BL-21 Strain)	(Laboratory Storage)	1.3 ×10^9^ CFU mL^−1^	10^−5^
4.	*Proteus vulgaris*	ATCC 6380 P	1.5 ×10^9^ CFU mL^−1^	10^−5^
5.	*Pseudomonas aeruginosa*	ATCC 15442	2.6 ×10^10^ CFU mL^−1^	10^−6^
6.	*Ralstonia insidiosa*	ATCC 49129	7.0 ×10^6^ CFU mL^−1^	10^−5^
7.	*Staphylococcus aureus*	ATCC 11632	1.7 ×10^10^CFU mL^−1^	10^−6^
8.	*Vibrio fischeri*	MTCC 1738	1.6 ×10^9^ CFU mL^−1^	10^−6^

### Validation of S-ELISA for WC detection of *Brucella* in different matrices

**Figure 3:**
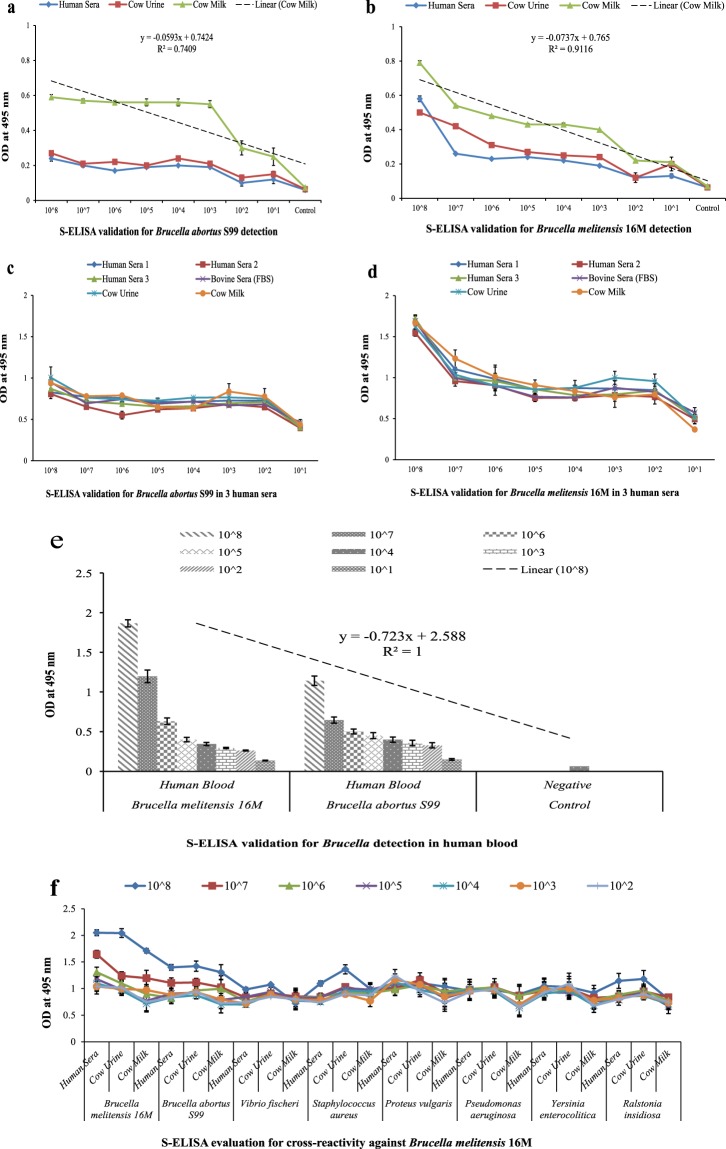
Validation of developed S-ELISA assay for detection of two *Brucella* spp. in different matrices spiked with 10 fold serial diluted WC Ag of *Brucella*. **(a,b)** S-ELISA graph plot showing detection in spiked matrices (WC Ag at 10^8^ CFU mL^−1^ to 10^1^ CFU mL^−1^) for *Brucella abortus* S99 and *Brucella melitensis* 16 M along x-axis and absorbance at 495 nm along y-axis respectively. Linear regression co-efficient value for initial validation in human sera, cow urine and cow milk is R^2^ = 0.7409 with linear model equation, y = - 0.0593×+ 0.7424 and, R^2^ = 0.9116 with linear model equation, y = - 0.0737×+ 0.765 for validation against *Brucella abortus* S99 and *Brucella melitensis* 16 M WC detection respectively. **(c,d)** S-ELISA graph plot showing detection in spiked matrices for *Brucella* with three different human sera and one foetal bovine sera for validation along x-axis and absorbance at 495 nm along y-axis for *Brucella abortus* S99 and *Brucella melitensis* 16 M WC detection respectively. **(e)** S-ELISA graph plot showing detection of *Brucella* spp. in spiked human whole blood along x-axis and absorbance at 495 nm along y-axis. Linear regression co-efficient value, R^2^ = 1 at no bias showing linearity with model equation, y = - 0.073×+ 2.588 for validation against *Brucella abortus* S99 and *Brucella melitensis* 16 M WC detection. OD value obtained is <1 at lower limits for both the spp. **(f)** S-ELISA graph plot showing detection in spiked matrices with 10 fold serial diluted WC Ag (10^8^ CFU mL^−1^ to 10^2^ CFU mL^−1^) of *Brucella melitensis* 16 M along with WC Ag of *Brucella abortus* S99 and closely *Brucella* related bacterial spp. for validation along x-axis and absorbance at 495 nm along y-axis for evaluation of cross-reactivity. Both *Brucella* spp. are cross-reactive on validation.

*Brucella* WC spiked clinical and non-clinical samples (Fig. 3a–d) were subjected for WC detection. It was found that on initial validation in different matrices, cow milk was showing more detection with detection limit of 10^3^ CFU mL^−1^ followed by urine and human sera at linear equation, y = − 0.0737×+0.765 with R^2^ = 0.9116 comparatively linear for *Brucella melitensis* 16 M WC detection and on further increasing the sample size with two more human serum and FBS, cow milk followed by urine and human sera 1 showed more detection for both the spp. comparative to rest of the clinical samples. Also, on test validation for optimized effective concentration of capture antibody at two different concentrations (10 and 20 µg mL^−1^) with *Brucella melitensis* 16 M WC spiked matrices presented standard curve of polynomial regression co-efficient having linear equation, y = − 0.002×^3^ + 0.057×^2^ − 0.500×+ 2.026 with R^2^ = 0.953 and, y = − 0.007×^3^ + 0.122×^2^ – 0.652×+ 1.438 with R^2^ = 0.911 for two concentrations (see Supplementary Fig. S4a,b online) respectively. The cow milk showed more detection followed by cow urine, human sera and bovine sera with maximum OD value ≥ 1 and R^2^ value near to 1 for best fit of standard curve at 10 µg mL^−1^ of optimized concentration and non-linear trend was obtained at 20 µg mL^−1^ concentration. The detection range obtained was 10^2^ to 10^8^ CFU mL^−1^ at an LOD of 10^3^ CFU mL^−1^. Also, on performing validation with *Brucella* WC spiked human whole blood as an important clinical sample for detection (Fig. 3e), a linear detection of R^2^ = 1 at equation, y = −0.723×+ 2.588 was obtained showing more detection with *Brucella melitensis* followed by *Brucella abortus* at lower detection limits of OD ≥ 0.5 respectively.

### Cross-reactive screening of S-ELISA for assay validation

For further validation of S-ELISA, an assay with *Brucella melitensis* 16 M along with *Brucella abortus* S99 and seven closely *Brucella* related spp. was test evaluated on similar spiked studies (Fig. 3f). It was observed that assay optimized was not cross-reactive with closely *Brucella* related spp. at detection limit and simultaneously, cross-reactive with *Brucella abortus* S99 at spp. level. Although, the assay was sensitive in detecting WC of *Brucella* spp. with LOD of 10^3^ CFU mL^−1^, further needs specificity for detection at spp. level.

### Immobilization of anti-*Brucella* mice IgG pAb on 4-MBA modified SPR-Au Chip

Immobilization of detection Ab depends on pH of the solution in which ligand was concentrated on 4-MBA modified SPR-Au Chip surface^37^. IgG pAb was pre-concentrated in neutral buffer, PBS (pH 7.2). Activation of biosensor chip by EDC/NHS carboxyl groups produces negative charge higher than the pH 3.5. Therefore, pre-concentration of Ab was done with buffer at pH 7.2 less than the ligand iso-electric point PI^38^. Suitable pH between PI of ligand and sensor surface pKa, facilitates ligand pre-concentration. Moreover, the PI of the Ab is 9 and EDC (for conventional amine coupling) requires uncharged amine groups. Therefore, neutral pH at value 7.2 results in efficient immobilization with noise-free enhanced signals on SPR^35^. In SPR, sensogram (see Supplementary Fig. S5a,b online) was showing nine major steps for immobilization of detection Ab raised against the two *Brucella* spp. respectively.

### Interaction study of *Brucella* WC with mice IgG pAb immobilized on 4-MBA modified SPR-Au Chip

**Figure 4:**
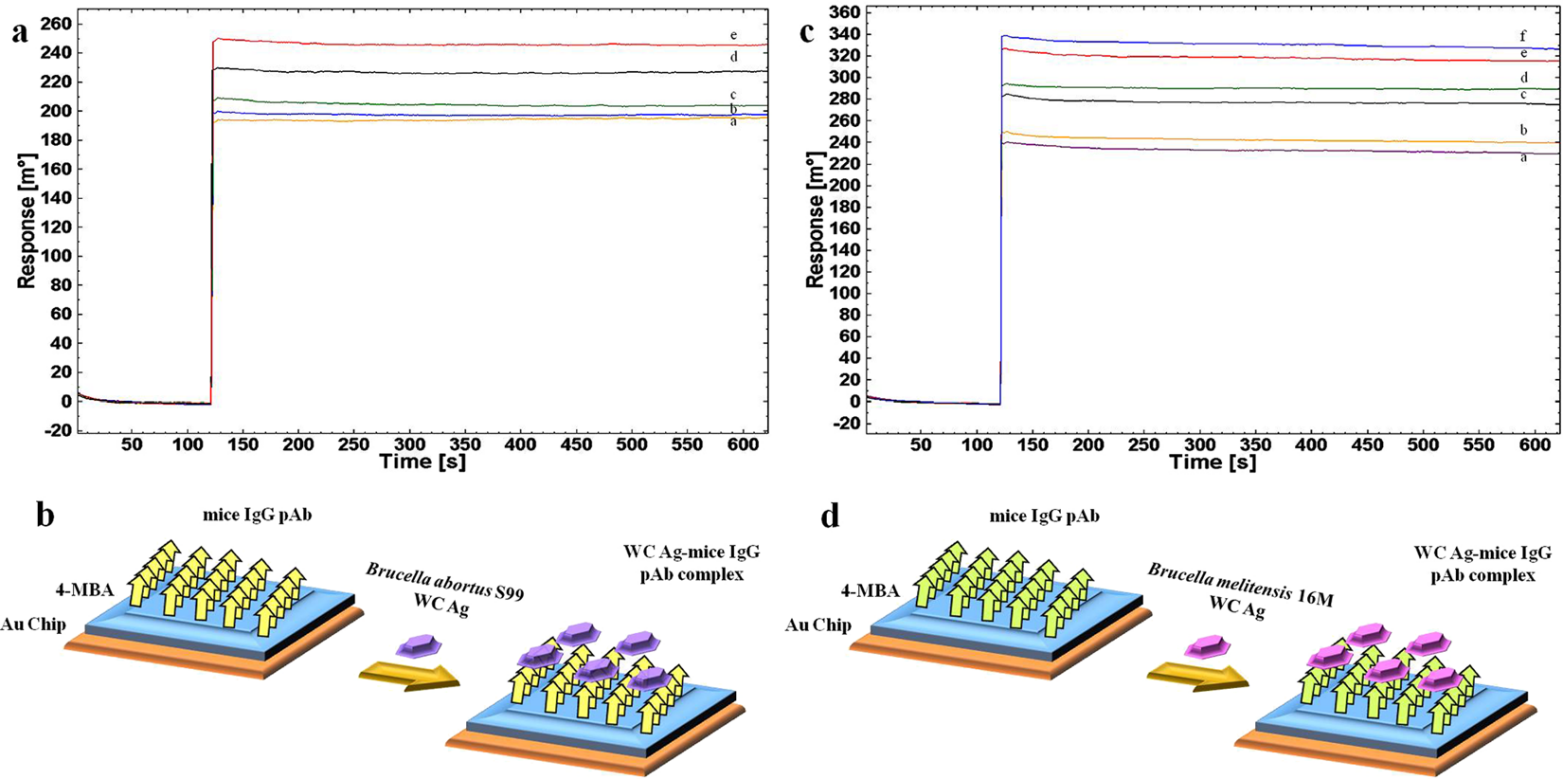
SPR sensor response and schematic illustration for interaction of immobilized mice IgG detection Ab with 10-fold serial diluted different concentrations of WC Ag of *Brucella* spp. **(a,b)** Interaction of detection Ab with WC Ag of *Brucella abortus* S99 at (a) 10^2^ CFU mL^−1^ (b) 10^3^ CFU mL^−1^ (c) 10^4^ CFU mL^−1^ (d) 10^5^ CFU mL^−1^ (e) 10^6^ CFU mL^−1^ and, **(c,d)** Interaction of detection Ab with WC Ag of *Brucella melitensis* 16 M, respectively at (a) 10^2^ CFU mL^−1^ (b) 10^3^ CFU mL^−1^ (c) 10^4^ CFU mL^−1^ (d) 10^5^ CFU mL^−1^ (e) 10^6^ CFU mL^−1^ (f) 10^7^ CFU mL^−1^ in PBS (pH 7.2) buffer at RT (25 °C) with time interval (in seconds) along x-axis and SPR response angle (m°) at y-axis for each interaction cycle.

Mice IgG pAb was immobilized on modified SPR-Au chip for sensing different concentration of *Brucella* WC (as analyte) for two *Brucella* spp. Sensogram for *Brucella abortus* S99 (Fig. 4a) and for *Brucella melitensis* 16 M (Fig. 4c) attributes to concentration-dependent interaction, gradually varied from 10^2^ to 10^6^ CFU mL^−1^ and 10^2^ to 10^7^ CFU mL^−1^ respectively. The schematic illustration for above interaction was shown (Fig. 4b,d) for both the spp. LOD, as to minimum concentration at which the response during interaction with immobilized Ab was measured and found to be 10^2^ CFU mL^−1^. Even on increasing the SPR probing depth (beyond 1 µm) employing long-range surface plasmons, dry mass of whole bacterium was measurable at minimum LOD observed for kinetic study^39,40^.

### Kinetic evaluation study of mice IgG detection pAb with cell envelope Ag of *Brucella*

Sensitivity for LOD of mice IgG pAb was evaluated against immobilized whole CE (Cell envelope surface) Ag of two *Brucella* spp. with 10 fold serial dilutions (10^2^ µg mL^−1^ to 10^−1^ pg mL^−1^). Limit of detection (LOD) was found to be 10^−1^ ng mL^−1^ (see Supplementary Fig. S6 a, b online) with linear SPR angle shift, detected at concentration 1 µg mL^−1^ to 10^−1^ ng mL^−1^. K_D_ value was calculated for binding interaction using data fitted into simple 1:1 interaction model, (A) + (B) = (AB), indicating (A) as analyte (mice IgG pAb) injected, (B) as CE Ag immobilized and (AB) as Ab-Ag complex (analyte-ligand) formed in SPR system during interaction. Hence, K_D_ value and B_max_ was found to be 16.48 pM and 81.67 m° for *Brucella abortus* S99 and 0.42 pM and 54.50 m° for *Brucella melitensis* 16 M, respectively with high affinity towards *Brucella melitensis* 16 M. The K_D_ value (<10 nM) signifies high affinity interaction among the complexes and signal R corresponds proportional to the amount of (AB) formed. Rmax is proportional to initial concentration of Ag (ligand) immobilized^41–43^.

### Serum-spiked validation for binding interaction of mice IgG pAb

**Figure 5:**
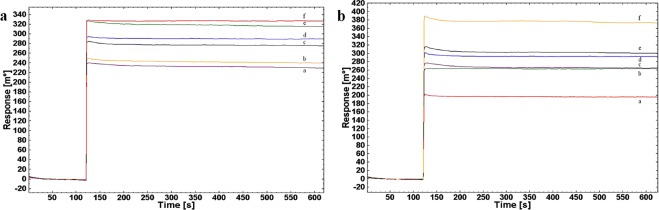
SPR sensor response for interaction of immobilized mice IgG detection Ab with 10-fold serial diluted serum-spiked different concentrations of *Brucella* WC Ag. **(a)** Interaction of detection Ab with serum-spiked WC Ag of *Brucella abortus* S99 at (a) 1:5000 dilution of serum in PBS as an experimental control (b) 10^2^ CFU mL^−1^ (c) 10^3^ CFU mL^−1^ (d) 10^4^ CFU mL^−1^ (e) 10^5^ CFU mL^−1^ (f) 10^6^ CFU mL^−1^ and, **(b)** Interaction of detection Ab with serum-spiked WC Ag of *Brucella melitensis* 16 M, respectively at (a) 1:5000 dilution of serum in PBS as an experimental control. (b) 10^2^ CFU mL^−1^ (c) 10^3^ CFU mL^−1^ (d) 10^4^ CFU mL^−1^ (e) 10^5^ CFU mL^−1^ (f) 10^6^ CFU mL^−1^ in FBS:PBS dilution (1:5000) at RT (25 °C).

*Brucella abortus* and *Brucella melitensis* intact WC (10^8^ to 10^1^ CFU mL^−1^) were spiked in serum (1:5000 of FBS in sterile PBS) samples to interact with immobilized mice IgG pAb (10 PPM) in real scenario. Ab-Ag binding avidity increases on increasing affinity between mice IgG pAb and WC Ag with number of specific interactions formed at each binding-regeneration cycle in response with SPR angle shift^44,45^. The SPR response of angle change (Fig. 5a,b) at concentration of 10^2^ to 10^6^ CFU mL^−1^ with LOD of 10^2^ CFU mL^−1^ was monitored with no effect of serum on interactions validated with non-spiked FBS as experimental control.

### SPR-biosensing assessment for co-operative binding of BSA protein

**Figure 6:**
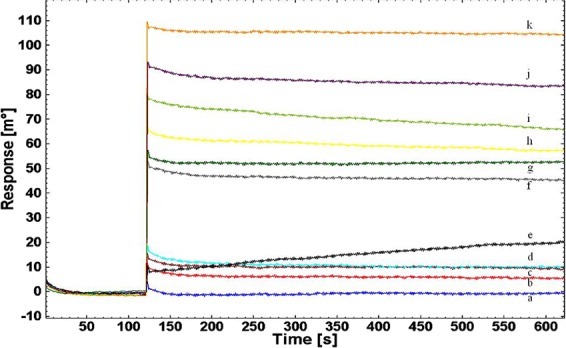
SPR sensor response for co-operative interaction of BSA protein along with 10-fold serial diluted different concentrations of WC Ag of *Brucella abortus* S99 on immobilized mice IgG detection Ab at (a) 10^−2^ µg mL^−1^ BSA (b) 10^−1^ µg mL^−1^ BSA (c) 1 µg mL^−1^ BSA (d) 10 CFU mL^−1^ e) 10 µg mL^−1^ BSA (f) 10^2^ CFU mL^−1^ (g) 10^3^ CFU mL^−1^ (h) 10^4^ CFU mL^−1^ (i) 10^5^ CFU mL^−1^ (j) 10^6^ CFU mL^−1^ (k) 10^7^ CFU mL^−1^ in PBS buffer at RT (25 °C).

Co-operative binding of BSA protein was studied along with either of WC Ag of *Brucella* spp. (*Brucella abortus* S99) on SPR. SPR response with angle shift was observed for BSA protein (10^−2^ µg mL^−1^ to 10 µg mL^−1^) and for *Brucella* WC Ag (10 CFU mL^−1^ to 10^7 ^CFU mL^−1^) on interaction with immobilized mice IgG detection Ab (Fig. 6). On evaluation, it was found that BSA protein exhibit non co-operative binding (below SPR response angle) with the ligand showing no interference in detection of *Brucella* WC Ag.

## Discussion

*Brucellosis* is an endemic and widespread zoonosis with a huge global burden of socio-economic importance and a potential biowarfare challenge to food and health security worldwide^46^. It mainly infects livestock and inhabiting human population with six potentially pathogenic species including *Brucella abortus*, *Brucella melitensis*, *Brucella suis*, *Brucella canis*, *Brucella ovis* and *Brucella neotomae*^47^. It has the ability to survive and multiply inside the host phagocytes causing febrile illness, undulant fever and significant reproductive in-efficiency like abortion and still-birth in cattle. The severity, pathogenicity and disease progression is a major call for new and improved treatment regimens to be provided in particular as a reliable tool for detection^48^. In this concern, we have developed an enzyme immunoassay which is detecting the intact WC of *Brucella* at sensitive representation in various matrices of clinical efficacy. *Brucella* shares maximum homology with type strains and similarity coverage is 98 to 99% at sub-type species level. Since, polyclonal antibody is reliable in potential capture of the antigen whereby we evaluated 10 µg mL^−1^ of WC rabbit IgG capture Ab as more sensitive in determining the lowest detection limits. Higher Ab-titer WC pAbs with an average ≥ 64,000 ranging upto 1,28,000 were used with no batch variations in optimizing the assay with linear detection range. Checker board S-ELISA provided multiple unique combinations of detection antibody and WC antigen concentrations evaluating the lower limit of detection for both test antibody and antigen respectively. The mice IgG detection Ab at 0.78 and 1.56 µg mL^−1^ concentration in checker board S-ELISA detected 10^3^ CFU mL^−1^ of *Brucella* WC experimentally with 10 µg mL^−1^ of rabbit IgG capture Ab as the lowest effective working concentration of detection antibody. Similarly, on characterization for the potential immuno-reactivity of WC IgG pAbs with both CE and SA Ag determined group 1 Omps of *Brucella* to be more reactive with mice IgG detection pAb. The outer membrane proteins of *Brucella* are highly antigenic surface proteins responsible in establishing the pathogenicity inside the host^11^. The detection Ab at concentration 50 µg mL^−1^ determined the cross-reactivity within *Brucella* spp. and no cross-reactivity with closely related bacterial spp. during interference study. The capture antibody at 10 µg mL^−1^ concentration also enabled sensitive linear detection of *Brucella* WC Ag as compared with 20 µg mL^−1^ concentration. Also, the optimized sensitive capture antibody on test validation detected *Brucella* WC Ag in cow milk followed by cow urine, human sera and bovine sera for both *Brucella* spp. at R^2^ value of 0.953 and similarly, detected *Brucella* in spiked human whole blood at R^2^ value of 1 with no bias in linear detection. Therefore, for detection of *Brucella* in blood and sera in relation to early acute infections, persisted infection in vaccinated and reservoir groups and in relapsed cases of the disease where WC detection of *Brucella* is preliminary, a potent and sensitive double antibody S-ELISA can be addressed significantly. In our study, we have also evaluated the experimental LOD of the developed immunoassay with sensitive SPR based bio-sensor in spiked and non-spiked samples at detection limit of 100 CFU with detection range of 10^2^ to 10^7^ CFU mL^−1^ for both *Brucella* spp. used in the study. The kinetic study determined the sensitivity of detection Ab on interaction with cell envelope Ag at a linear shift of detection from 1 µg to 10^−1^ ng mL^−1^ of antibody at K_D_ value of 16.48 and 0.42 pM for *Brucella abortus* S99 and *Brucella melitensis* 16 M respectively. The K_D_ value (<10 nM) represents high affinity interaction which is comparatively more towards *Brucella melitensis* 16 M WC detection. This study also relatively evaluated the efficacy of immunoassay experimentally at lowest detection limits, below 1 µg mL^−1^ for detection antibody at concentration ranging from 100 µg mL^−1^ to 1.56 and 0.78 µg mL^−1^ for two *Brucella* spp. respectively. Most of the immunoassay shows cross-reaction with inevidently characterized surface antigens which we attempted for validation using cross-reactive studies and revealed that both the *Brucella* spp. are cross-reacting with each other at spp. level due to similarity between sub-types^49^. No cross-reactivity was observed with other bacterial spp. at optimized concentration of capture and detection antibody. For further detection in human sera and whole blood, spiked WC Ag of *Brucella* was detected for evaluation of immunoassay in SPR and with S-ELISA at LOD of 10^2^ and 10^3^ CFU mL^−1^ respectively. For biological interference study on affinity interactions in SPR, co-operative binding of BSA protein resulted in no relative interference and *Brucella* WC Ag was detected at 10^1^ to 10^7^ CFU mL^−1^. This study therefore, validated the detection of WC in different important samples in perspective to *Brucella* infection by applying bio-sensor and immuno-assay based platforms to evaluate the effective antigen-antibody interactions in concentration-dependent manner. Thus, *Brucella* WC detection using selective and sensitive set of double-antibody combination in S-ELISA format is potentially reliable and can be employed for detection at genus level as demonstrated for the use in various clinical and environment matrices.

## Conclusion

Our study indicated that pAb can detect whole bacterial cell with increased sensitivity and IgG pAb specific to *Brucella* captures WC bacteria with maximum affinity. We developed and validated the WC based sandwich-ELISA for detection of *Brucella abortus* and *Brucella melitensis* with LOD upto 10^3^ CFU mL^−1^. Moreover, it was found that both *Brucella abortus* S99 and *Brucella melitensis* 16 M were cross-reacting with each other at spp. level within the detection assay. The Polyclonal IgG Ab against WC Ag of *Brucella abortus* S99 was able to detect *Brucella melitensis* and vice-versa. It was therefore, concluded that the developed immunoassay was sensitive in detecting whole bacterial cell of *Brucella*. Also, Immunoblot studies using mice IgG detection pAb revealed that the characterized proteins belong to group 1 (94 or 88 kDa) minor immunogenic OMPs of *Brucella* which are known to be potentially immunogenic. Further, on kinetic evaluation using SPR, K_D_ value calculated for *Brucella abortus* S99 was 16.48 pM and for *Brucella melitensis* 16 M was 0.42 pM respectively, measuring high affinity towards *Brucella melitensis* 16 M for the developed assay. Thus, SPR based biosensor detected WC bacteria with increased sensitivity at LOD of 10^2^ CFU mL^−1^. Despite, SPR being more sensitive and accurate in determining the Ag-Ab interactions this may not be preferred for field deployment directly due to high cost, whereas the workability of developed S-ELISA can be deployed as an immunoassay for kit based detection system for *Brucella*. Therefore, upon validation of S-ELISA (as an ancillary diagnostic immunoassay for stability tests and performance evaluation) with spiked studies in clinical and non-clinical samples evaluated linear detection in cow’s milk followed by urine and human sera for both *Brucella abortus* S99 and *Brucella melitensis* 16 M. We therefore, concluded in our study that the developed semi-quantitative S-ELISA is potent, rapid and sensitive in detecting intact-whole bacterial cell of *Brucella*. And, in future, can be made more specific by replacing detection Ab with mAb specific to recombinant Ag of *Brucella* spp. (specifically OMP derived) with minimum cross-reactivity and maximum specificity for the optimized available immunoassay.

## Methods

### Chemicals and Reagents

*Brucella* Selective Broth (BSB) and Agar (Hi-media), Polyclonal goat and rabbit anti-mice immunoglobulins/HRP, Polyclonal goat anti-rabbit immunoglobulins/HRP (Dako-Denmark), O-Phenylenediamine dihydrochloride (OPD) from Sigma-Aldrich, 3,3’-Diaminobenzidine (DAB) Sigma-Fast Tablets, Protein-A antibody purification kit (Montage-Millipore, USA). Pure analytical grade chemicals for SPR work from Sigma-Aldrich (Fluka) and Merck as; N-(3-dimethylaminopropyl)-N’-ethylcarbodiimide hydrochloride (EDC), N-hydroxysuccinimide (NHS), 4-Mercaptobenzoic acid (4-MBA), Ethanolamine, Heparin (Hi-media), Sodium Azide, Methanol (MeOH), Phosphate Buffered Saline (PBS, pH 7.2, 10 mM L^−1^) and Hydrochloric acid (0.01 M HCl).

### Instruments and Apparatus

EUTECH Thermo pH-meter and ultrapure Milli-Q water (Millipore). µQuant BioTek ELISA reader for protein estimation and ELISA. Hoefer TE22 Amersham Biosciences Western Blot transfer unit and BIO-RAD Mini-PROTEAN Tetra Cell for SDS-PAGE. SPR-Au chip (XanTec Bioanalytics GmbH, Metrowingerplatz, Germany) was modified with 4-MBA using spin coater (Autolab Spin Coater, Eco Chemie B.V., Utrecht, The Netherlands). A double-channel cuvette based electrochemical SPR system (Autolab ESPIRIT, Eco Chemie B.V., The Netherlands), where diode laser was a fixed wavelength source at 670 nm. One channel utilized for SPR assay and second as reference channel maintained at a fixed temperature with Julabo HE-4 water bath (Julabo Labortechnik GmbH, Seelbach, Germany). Data acquisition software (version 4.3.1.) and kinetic evaluation software (version 5.0) provided automated SPR real-time monitoring and kinetics.

### Ethical Approval

This work was carried out at Defence Research and Development Establishment, DRDO, Ministry of Defence, Government of India and approved by Institutional Animal Ethics Committee (No: 37/GO/Rbi/S/99/CPCSEA) for purpose of control and supervision of experimental animals. All the methods were carried out in accordance with the relevant guidelines and regulations. This study is also approved by Institutional Biosafety Committee of Defence Research and Development Establishment, DRDO, Ministry of Defence, Government of India vide protocol no. IBSC/15/MB/DTS/6.

### Bacterial strains

Bacterial strains (Table 1) routinely cultured and maintained (30% glycerol at −80 °C) in our laboratory were used.

### Preparation of whole cell (WC) Ag and animal immunization

Pure colony culture in 5 mL broth (BSB) was incubated at 37 °C for 24 hrs at 180 rpm on shaker incubator (Labcon 5081U from Labcon, USA). Serial dilutions (10^−10^ to 10^−1^ mL^−1^) were prepared for CFU count mL^−1^. Chemically inactivated the culture (1% HCHO for 1 hr) and centrifuged at 10,000 rpm for 20 min (at Room Temperature, RT). Pellet re-suspended in 5 mL PBS (1X) and aliquoted as *Brucella* spp. WC Ag. Similarly, WC Ag was prepared for closely related bacterial spp. BALB/c female mice and White New Zealand female rabbit from animal house facility of DRDE laboratory were immunized (1 ×10^5^ CFU and 1 ×10^9^ CFU respectively) with WC Ag of *Brucella* spp. as per protocol^50–52^.

### Production of pAbs and IgG purification

Whole blood collected from immunized animals was incubated at 37 °C for 1 hr and centrifuged at 7,000 rpm for 10 min. at 4 °C. One part of 1 M Tris-HCl (pH-8.0) was added to ten parts of supernatant and saturated (100%) ammonium sulphate solution (SAS, Sigma) was added upto 50% saturation. Centrifuged (as above), after stirring for 1 hr at 4 °C. Washed pellet with equal volumes of SAS at 10,000 rpm for 20 min (at 4 °C) and albumin removed by IPA-TCA method followed by re-suspension in 10X PBS for dialysis against 1X PBS (overnight, O/N at 4 °C)^53^. Dialysed samples were affinity purified with Montage protein-A columns. Total yield obtained was 2 to 6 mg mL^−1^ (batch-wise by Lowry method) and analysed by SDS-PAGE^54,55^. Aliquoted pAb was stored at -20 °C until use.

### WC based indirect ELISA (I-ELISA) for antibody titer

WC Ag (1.2 ×10^8^ CFU mL^−1^) was immobilized on ELISA immuno-modules (Thermo-Nunc F8 Maxisorp) as per protocol^56,57^. Washed with PBS/PBS-T thrice and blocked with 1% Bovine Serum Albumin (BSA, Hi-media) O/N at 4 °C. Titrated with 2 fold dilution of mice and rabbit anti-serum and incubated at 37 °C for 1 hr. Washed and incubated at 37 °C for 1 hr with 100 µL of rabbit anti-mice and goat anti-rabbit (1:1000) IgG/HRP conjugate, respectively. Washed and developed with OPD and H_2_O_2_ in 0.1 M Citrate Phosphate Buffer (pH-5.2) for 5 minutes. Ten µL of stop solution (1 N H_2_SO_4_) was added per well and absorbance was measured at 495 nm on ELISA reader. High Containment Facility (DRDE-DRDO, Gwalior, India) for experiments was used to avoid aerosolization.

### SDS-PAGE and western blot analysis of purified pAbs

Purified pAbs developed against WC Ag of both *Brucella* spp. at 10 µg of protein concentration were applied on 12% SDS-PAGE for analysis and characterized by Immuno-blotting. Ten µL of heat-treated and WC Sonicated (SA) Ag at 5 µg protein concentration per antigen was applied for SDS gel electrophoresis (Heat-treated or boiled Ag was prepared by boiling bacterial pellet in 2X of lysis buffer). WC, SA and CE Ag were prepared with total yield of 1 to 2 mg mL^−1^ protein as per protocol^58^. Transferred gel at 4 °C to Nitrocellulose Membrane (Thermo) in Tris-glycine buffer (pH 8.3) at a constant current of 2 mA cm^−2^. Membrane blocked in 5% of Skimmed Milk Powder (O/N at 4 °C). Washed and incubated (at RT for 1 hr) with mice IgG pAb (1:100) for two *Brucella* spp. respectively. Washed and incubated (at RT for 1 hr) with rabbit anti-mouse IgG/HRP conjugate (1:500). Washed and developed in DAB and H_2_O_2_ prepared in distilled water (DW). Reaction was stopped using DW.

### WC based S-ELISA for *Brucella* detection

Rabbit IgG pAb (capture Ab, 10 µg mL^−1^) was coated (at 100 µL per well) for two *Brucella* spp. and incubated O/N at 4°C). Washed and blocked (as above) and further, incubated at 37 °C for 1 hr with 1.2 ×10^8^ CFU mL^−1^ of WC Ag respectively. Washed and incubated at 37 °C for 1 hr with 2 fold diluted 100 and 200 µg mL^−1^ of mice IgG pAb (at 100 µL) as detection Ab. Washed and incubated at 37 °C for 1 hr with rabbit anti-mice IgG/HRP conjugate (1:1000 at 100 µL). Finally, assay was developed (as above in I-ELISA). Absorbance was measured at 495 nm.

### Determination of Detection range and LOD for WC S-ELISA

Checker-board S-ELISA was performed whereby, ELISA plates were coated in duplicates and blocked (as above in S-ELISA assay) for two *Brucella* spp. respectively and incubated at 37 °C for 1 hr with 10 fold serial dilution of *Brucella* WC Ag (10^8^ CFU mL^−1^ to 10^1^ CFU mL^−1^) at 100 µL. Washed and incubated at 37 °C for 1 hr with 2 fold serially diluted (100 µg mL^−1^ to 0.19 µg mL^−1^) mice IgG pAb and pre-immunization sera (mice PIS) as a negative control (1:1000 at 100 µL). Washed and incubated at 37 °C for 1 hr with rabbit anti-mice IgG/HRP conjugate (1:1000). Washed and developed (as above in I-ELISA). Absorbance was measured at 495 nm.

### Interference study for cross-reactivity

*Brucella abortus* and *Brucella melitensis* along with closely related bacteria (Table 1) were subjected for detection with developed S-ELISA (as above). Whereby, 10 fold serial dilution of WC Ag (10^8^ CFU mL^−1^ to 10^1^ CFU mL^−1^) was detected by 2 fold serially diluted mice IgG pAb (at 100 µg mL^−1^ to 25 µg mL^−1^) respectively and absorbance was measured at 495 nm.

### Validation of S-ELISA for WC detection of *Brucella* in different matrix

WC Ag of two *Brucella* spp. (10^8^ to 10^1^ CFU mL^−1^) were spiked in different matrix for detection using S-ELISA (with capture Ab at 10 µg mL^−1^ and detection Ab at 50 µg mL^−1^) as above. Whereby, clinical samples were healthy human whole blood and serum from DRDE (1:1000), foetal bovine serum (FBS at 1:1000) and morning mid-stream bovine urine (1:1 in PBS). Unpasteurized healthy bovine morning raw-milk (1:1 in PBS) as non-clinical sample was used (collected from local dairy of Gwalior, India). Absorbance was measured at 495 nm.

### Validation and cross-reactive screening of S-ELISA with *Brucella* spp

For further validation and evaluation of cross-reactivity, S-ELISA was performed for *Brucella melitensis* 16 M along with *Brucella abortus* S99 and closely related bacterial spp. by spiking (at 10^8^ to 10^1^ CFU mL^−1^) similarly in different matrix (as above). Rabbit IgG pAb (capture Ab at 10 µg mL^−1^) and mice IgG pAb (detection Ab at 50 µg mL^−1^) raised against WC Ag of *Brucella melitensis* 16 M were used and absorbance was measured at 495 nm.

### SPR kinetic evaluation study for pAb based affinity investigation of *Brucella*

In this study, bio-molecular interactions were evaluated with detection Ab (mice IgG pAb) for measuring the LOD, K_D_ and B_max_ values using SPR.

### Modification of SPR-Au chip as a bio-receptor functionalized with 4-MBA

SPR-Au chips were modified with 0.01 M methanolic solution of 4-MBA using spin coater as reported earlier^18^.

### Immobilization of anti-*Brucella* detection Ab and CE Ag on modified SPR-Au chip

For baseline stabilization, 50 µL PBS buffer was allowed to pass through the channels at every 120 s interval for 600 s. Modified SPR-Au chip surface was chemically activated by injecting 75 µL (300 s) of carboxyl activating agents, EDC (400 mM) and NHS (100 mM) mixture (1:1 ratio). Conventional primary-amine coupling with amine-active NHS esters facilitates amide bond formation with proteins. Seventy-five µL of mice IgG pAb (detection Ab) at 10 PPM (10 µg mL^−1^) was injected for immobilization in SPR channel (900 s) and 75 µL of PBS buffer was injected in reference channel on SPR Au-chip (900 s). Non-reacted surface active free sites were blocked by injecting 75 µL of 1000 mM (1 M) Ethanolamine (pH-8.5) at 600 s. Ten mM HCl was injected for surface regeneration. Similarly, 75 µL of CE Ag (10 PPM) was also immobilized on SPR-Au chip.

### SPR bio-sensing protocol

In an automated process, baseline stabilization was followed by interactive association (500 s), dissociation (400 s) and surface regeneration (120 s). Seventy-five µL of sample (analyte) was injected automatically into double channel SPR system from 384 well (24 ×16 well) microtiter plate and mixed at flow rate of 16.7 µL s^−1^ with series of such analyte acquisition repeated for each cycle. PBS as running buffer was used in channel washing. Analyte interaction on modified SPR-Au chip immobilized with mice IgG pAb was performed using inactivated WC Ag (10^8^ to 10^1^ CFU mL^−1^) of both *Brucella* spp. respectively. Interaction curve of binding (at each binding-regeneration cycle) was evaluated to study the concentration parameter. And, kinetic evaluation of mice IgG pAb as analyte (100 µg mL^−1^ to 10^−1^ pg mL^−1^) was performed on interaction with immobilized CE Ag of *Brucella* to study the kinetic parameter of SPR response at equilibrium.

Similarly for real scenario, WC Ag of *Brucella* spp. was spiked in FBS (1:5000) and interacted with immobilized mice IgG pAb for measuring LOD. Seventy percent of serum albumin may elicit co-operative binding during SPR detection. Therefore, 10 fold dilution of BSA (100 µg mL^−1^ to 1 ng mL^−1^) along with WC Ag of *Brucella abortus* S99 (10^8^ to 10^1^ CFU mL^−1^) was interacted with immobilized mice IgG (10 PPM) pAb against *Brucella abortus* S99 to study the albumin interference.

(Data not shown is available as supplementary file and data sets along with the manuscript).

## Supplementary information

Supplementary Information.

Dataset 1.
