# Supplementary material for: Development and validation of immunoassay for whole cell detection of Brucella abortus and Brucella melitensis
Source: Sci Rep. 2020 May 22;10:8543. doi: 10.1038/s41598-020-65347-9 (PMC7244763; doi:10.1038/s41598-020-65347-9)
Supplement: Supplementary file 1 — Supplementary Information. [file 41598_2020_65347_MOESM1_ESM.pdf]

Development and validation of immunoassay for whole cell detection of *Brucella abortus* and *Brucella melitensis*

Richa Hans, Pranjal Kumar Yadav, Pushpendra Kumar Sharma, Mannan Boopathi, Duraipandian Thavaselvam*

Defence Research and Development Establishment, DRDO, Jhansi Road, Gwalior - 474002, India.

*Corresponding author:

Tel.: +917512390331, Fax: +917512341148

Email address: dtselvam@drde.drdo.in

Supplementary Information

(Includes supplementary figures, graphs and legends)

Supplementary Figure S1. Whole cell (WC) based indirect ELISA (I-ELISA) for Ab titer estimation.

(a) WC based I-ELISA for determining mice hyper immune sera (HIS) antibody titer against WC Ag of *Brucella abortus* S99.

(a)

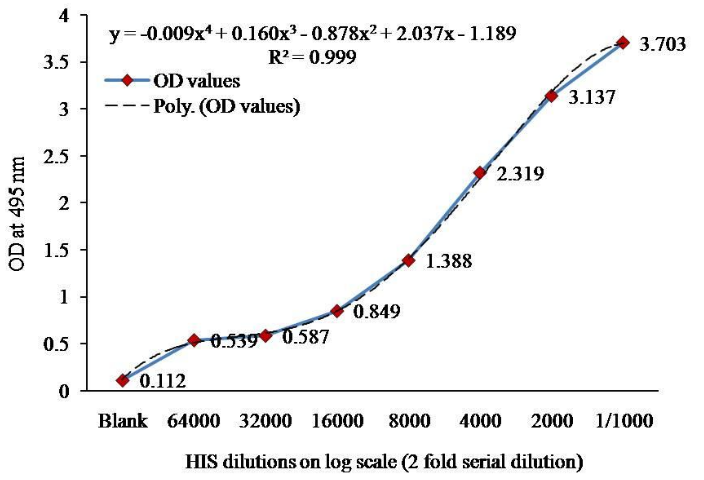

(b) WC based I-ELISA for determining rabbit hyper immune sera (HIS) antibody titer against WC Ag of *Brucella abortus* S99.

(b)

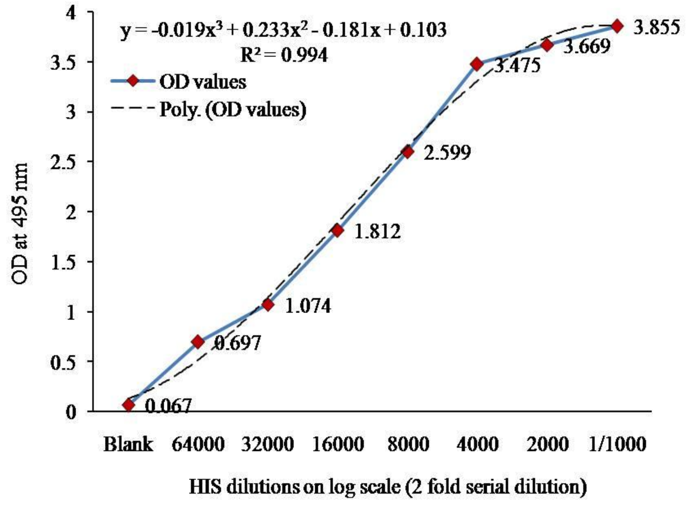

(c) WC based I-ELISA for determining mice hyper immune sera (HIS) antibody titer against WC Ag of *Brucella melitensis* 16M.

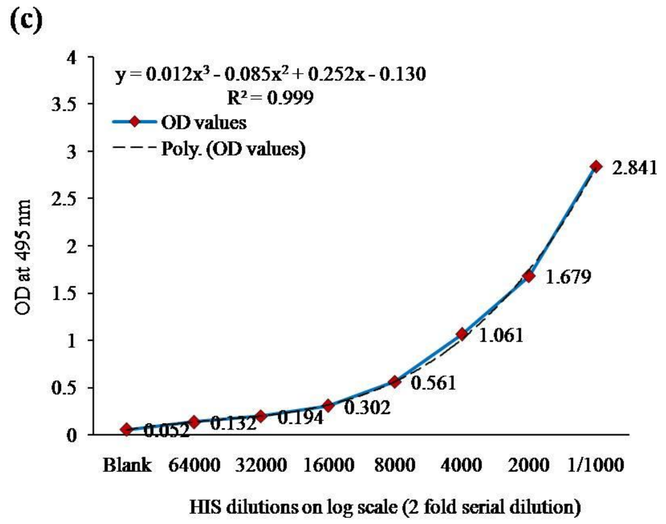

(d) WC based I-ELISA for determining rabbit hyper immune sera (HIS) antibody titer against WC Ag of *Brucella melitensis* 16M.

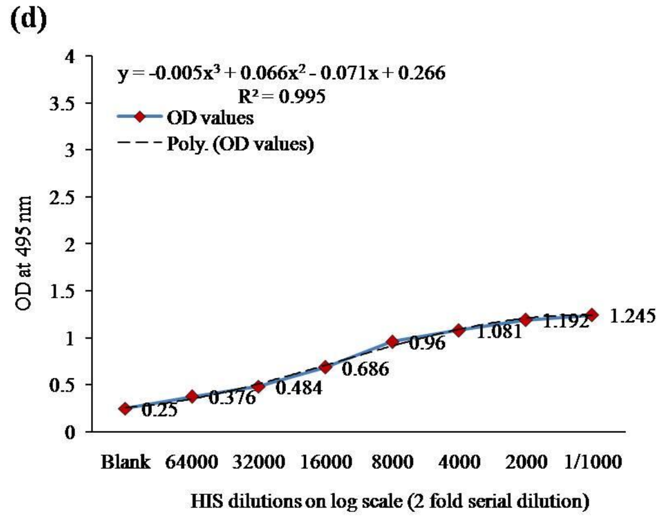

Supplementary Figure S2. SDS PAGE analysis of purified rabbit and mice IgG pAbs developed against WC Ag of two *Brucella* species (spp.).

(a) Conventional SAS purified rabbit IgG pAb against WC Ag of *Brucella abortus* S99 and *Brucella melitensis* 16M (Lane 1 and 2) and mice IgG pAb against WC Ag of *Brucella*

abortus S99 and *Brucella melitensis* 16M (Lane 3 and 4), Lane M with Fermentas #SM0671 protein marker.

(a)

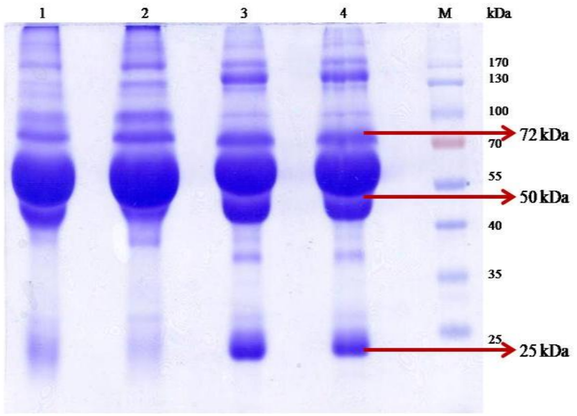

(b) Column purified (albumin removed) rabbit IgG pAb against WC Ag of *Brucella abortus* S99 and *Brucella melitensis* 16M (Lane 1 and 2) and mice IgG pAb against WC Ag of *Brucella abortus* S99 and *Brucella melitensis* 16M (Lane 3 and 4), Lane M with Fermentas #SM0671 protein marker.

(b)

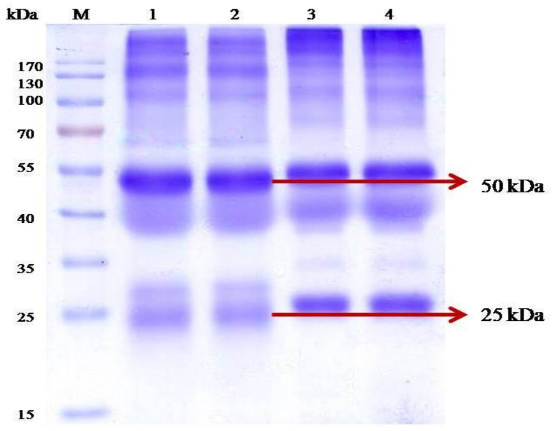

Supplementary Figure S2. SDS PAGE analysis of whole cell sonicated (SA) and cell envelope (CE) Ag of *Brucella* spp.

(c) SA Ag of *Brucella melitensis* 16M (Lane 1 and 2) and *Brucella abortus* S99 (Lane 3 and 4) respectively. Lane M with Fermentas #SM0671 protein marker.

(c)

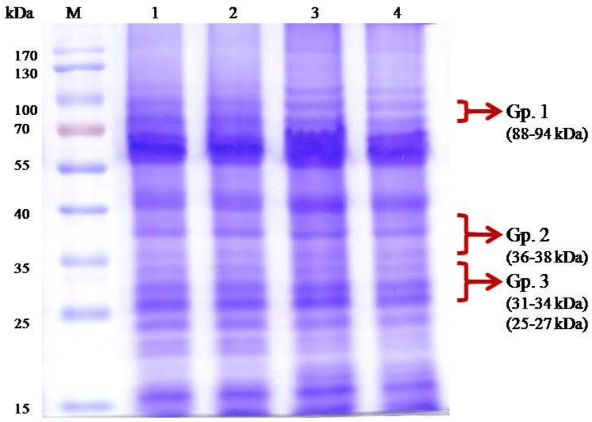

(d) CE Ag of *Brucella melitensis* 16M (Lane 1 and 2) and *Brucella abortus* S99 (Lane 3 and 4) respectively. Lane M with Fermentas #SM0671 protein marker.

(d)

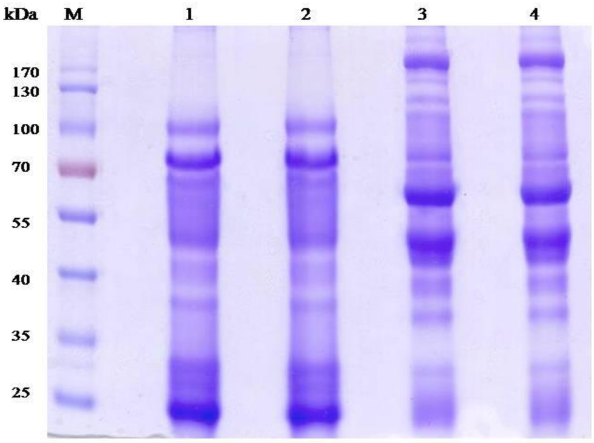

Supplementary Figure S2. Western blot characterization of different *Brucella* antigens with WC mice IgG detection pAb.

(e) SA Ag of *Brucella abortus* S99 and *Brucella melitensis* 16M (Lane 1 and 2) with their immunoblot (Lane 1 and 2) respectively. Lane M with Fermentas #SM0671 protein marker.

(e)

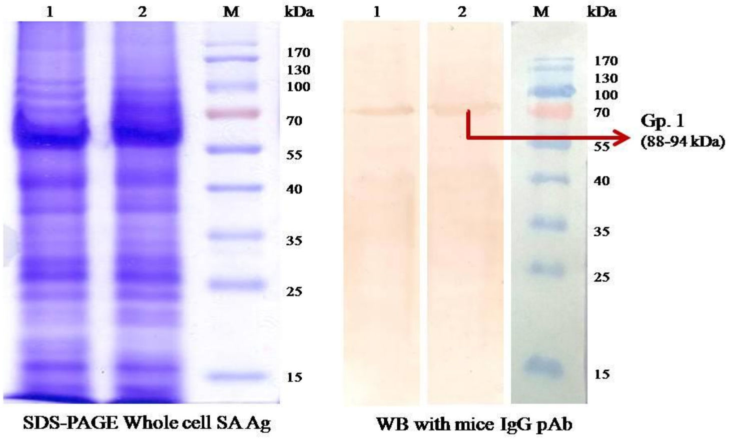

(f) Whole cell heat treated (heat-boiled) Ag of *Brucella melitensis* 16M (Lane 1 and 2) and *Brucella abortus* S99 (Lane 3 and 4) with their immunoblot (Lane 1 and 2) respectively. Lane M with Fermentas #SM1811 protein marker.

(f)

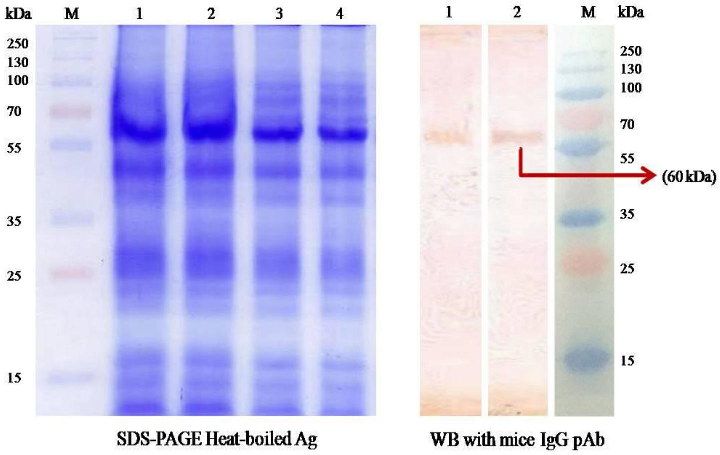

Supplementary Figure S3. Whole cell based sandwich ELISA (S-ELISA) assay for detection of *Brucella* spp.

(a) S-ELISA assay for the detection of *Brucella abortus* S99 WC Ag with rabbit IgG pAb as capture Ab and mice IgG pAb (at 2 fold serial dilution) as detection Ab.

(a)

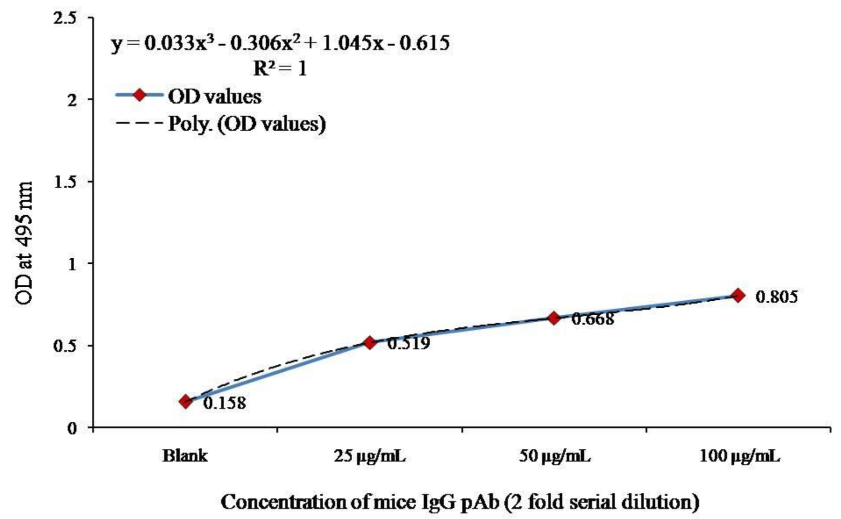

(b) S-ELISA assay for the detection of *Brucella melitensis* 16M WC Ag with rabbit IgG pAb as capture Ab and mice IgG pAb (at 2 fold serial dilution) as detection Ab.

(b)

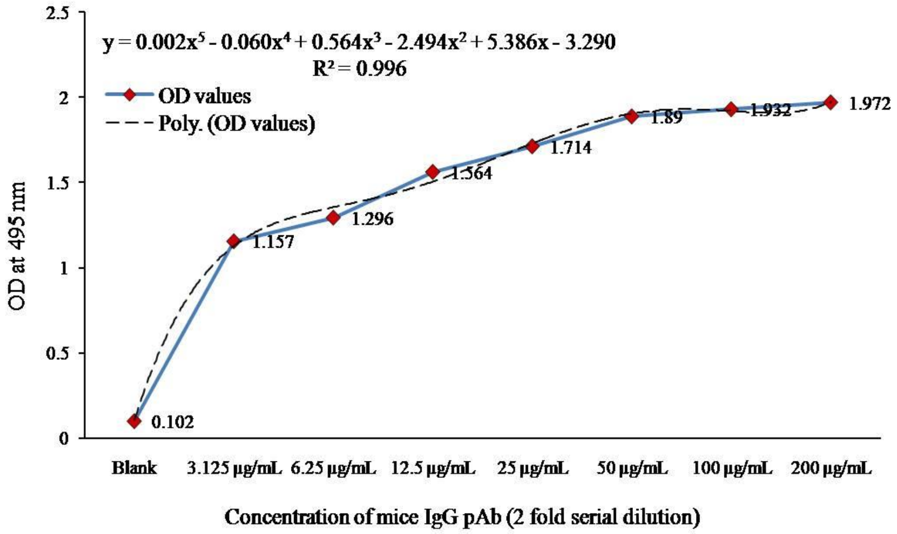

Supplementary Figure S4. Test validation of developed S-ELISA assay at two different concentrations of WC rabbit IgG capture pAb for *Brucella melitensis* 16M WC detection.

(a) Test validation with spiked WC Ag of *Brucella melitensis* 16M in different matrices using S-ELISA at 10 µg mL^{-1} of rabbit IgG capture Ab and 50 µg mL^{-1} of mice IgG detection Ab against *Brucella melitensis* 16M WC Ag.

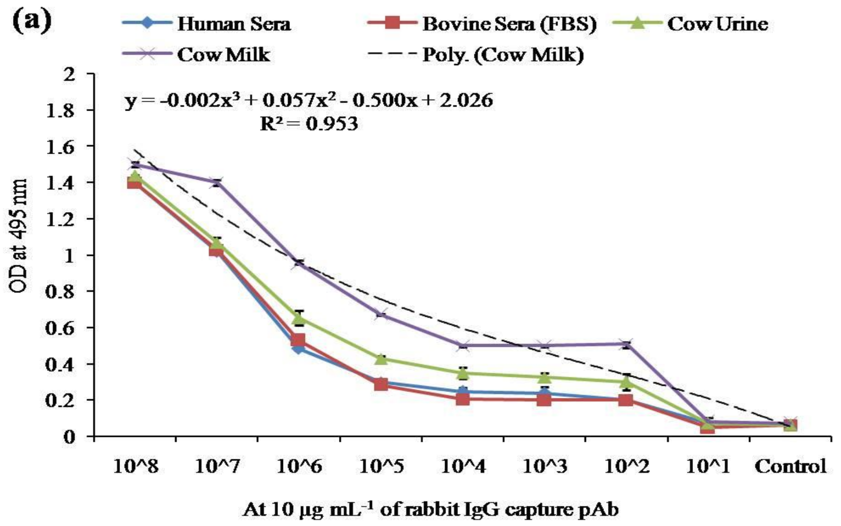

(b) Test validation with spiked WC Ag of *Brucella melitensis* 16M in different matrices using S-ELISA at 20 µg mL^{-1} of rabbit IgG capture Ab and 50 µg mL^{-1} of mice IgG detection Ab against *Brucella melitensis* 16M WC Ag.

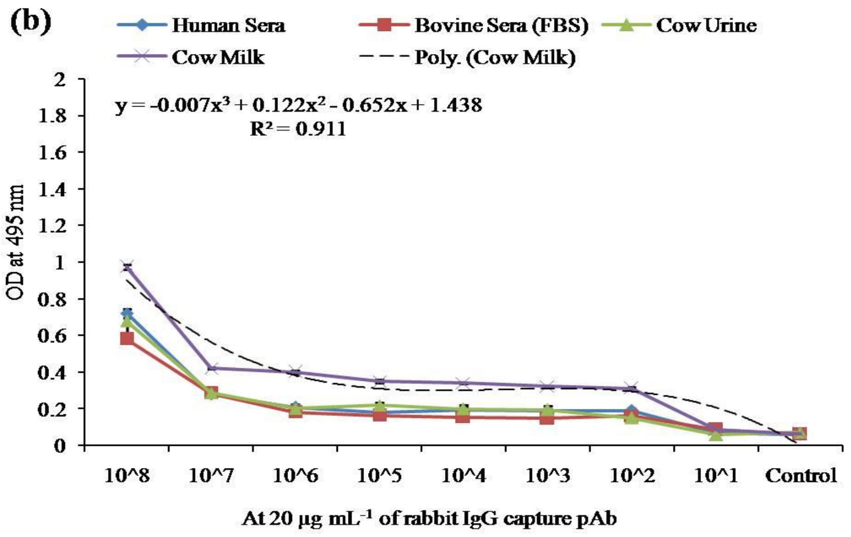

Supplementary Figure S5. Sensogram of SPR showing different steps for immobilization of mice IgG detection pAb.

(a) Immobilization of mice IgG pAb developed against WC Ag of *Brucella abortus* S99 (from left to right) respectively on 4-MBA modified SPR-Au chip surface.

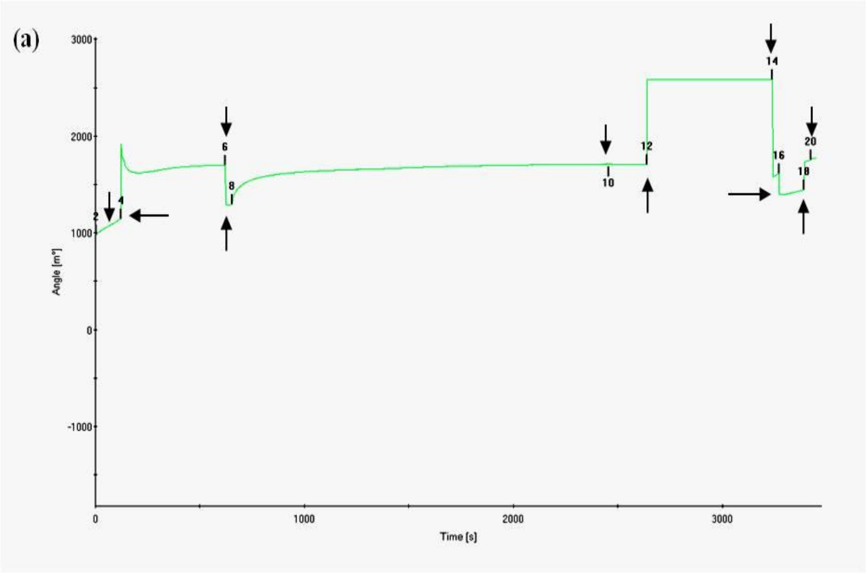

(b) Immobilization of mice IgG pAb developed against WC Ag of *Brucella melitensis* 16M (from left to right) respectively on 4-MBA modified SPR-Au chip surface.

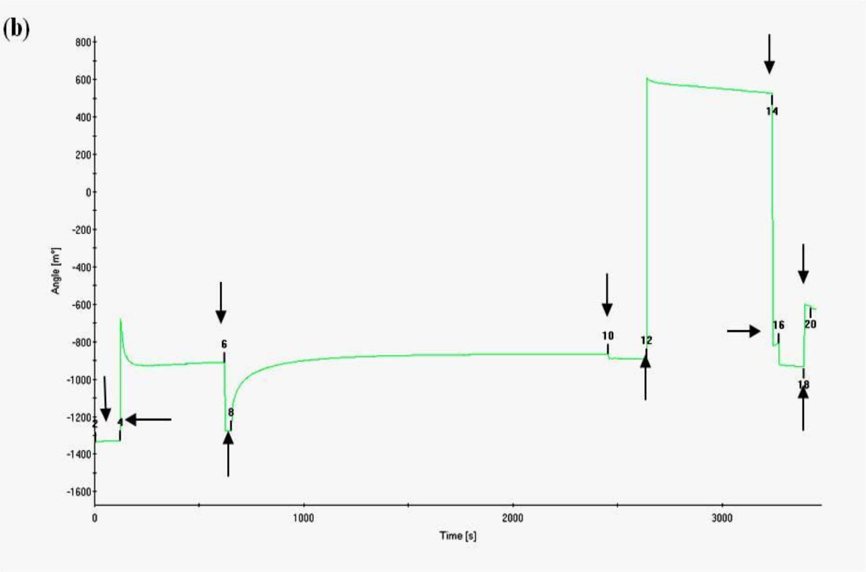

Supplementary Figure S6. SPR sensor response for interaction of immobilized whole CE Ag of *Brucella* with different concentrations of mice IgG detection pAb.

(a) SPR sensor response for interaction of immobilized *Brucella abortus* S99 whole CE Ag with different concentrations of mice IgG detection Ab (a) 10^{-1} ng mL^{-1} (b) 1 ng mL^{-1} (c) 10^{-2} µg mL^{-1} (d) 10^{-1} µg mL^{-1} (e) 1 µg mL^{-1} in PBS.

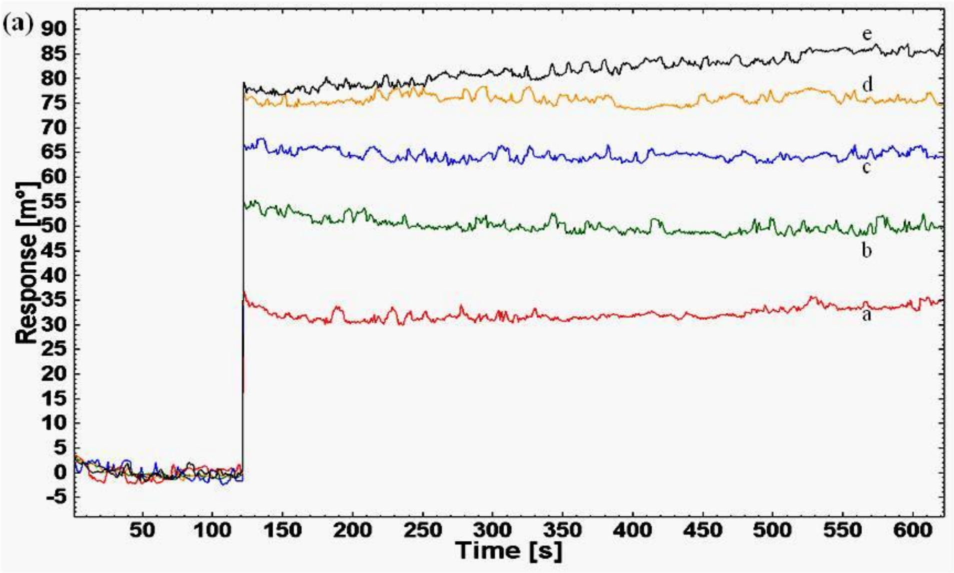

(b) SPR sensor response for interaction of immobilized *Brucella melitensis* 16M whole CE Ag with different concentrations of mice IgG detection Ab (a) 10^{-1} ng mL^{-1} (b) 1 ng mL^{-1} (c) 10^{-2} µg mL^{-1} (d) 10^{-1} µg mL^{-1} (e) 1 µg mL^{-1} in PBS.

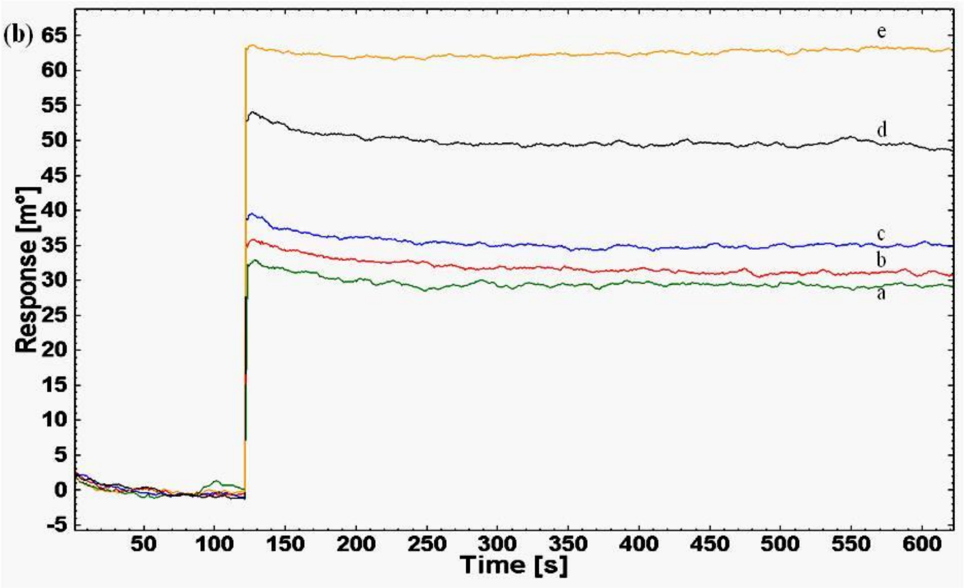

Supplementary Figure S6. SPR sensor response at equilibrium for interaction of immobilized CE Ag of *Brucella* spp. with different concentration of mice IgG detection pAb at temperature - 25 °C and pH - 7.2 for evaluation of kinetic parameters.

(c) SPR sensor response at equilibrium for interaction of immobilized CE Ag of *Brucella abortus* S99 with different concentration of mice IgG detection Ab for kinetic evaluation.

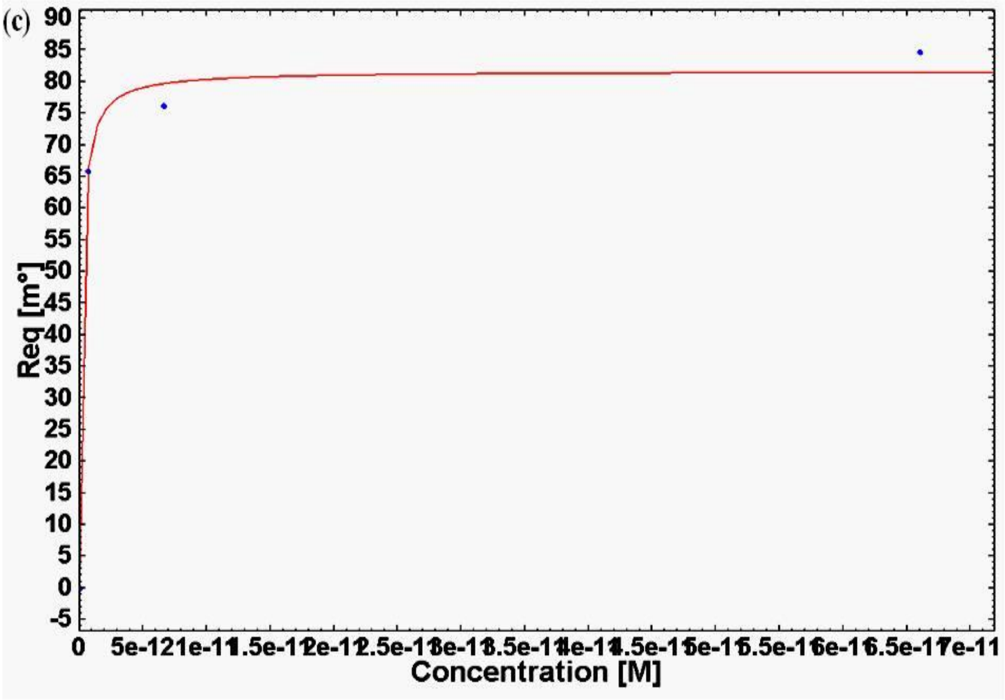

(d) SPR sensor response at equilibrium for interaction of immobilized CE Ag of *Brucella melitensis* 16M with different concentration of mice IgG detection Ab for kinetic evaluation.

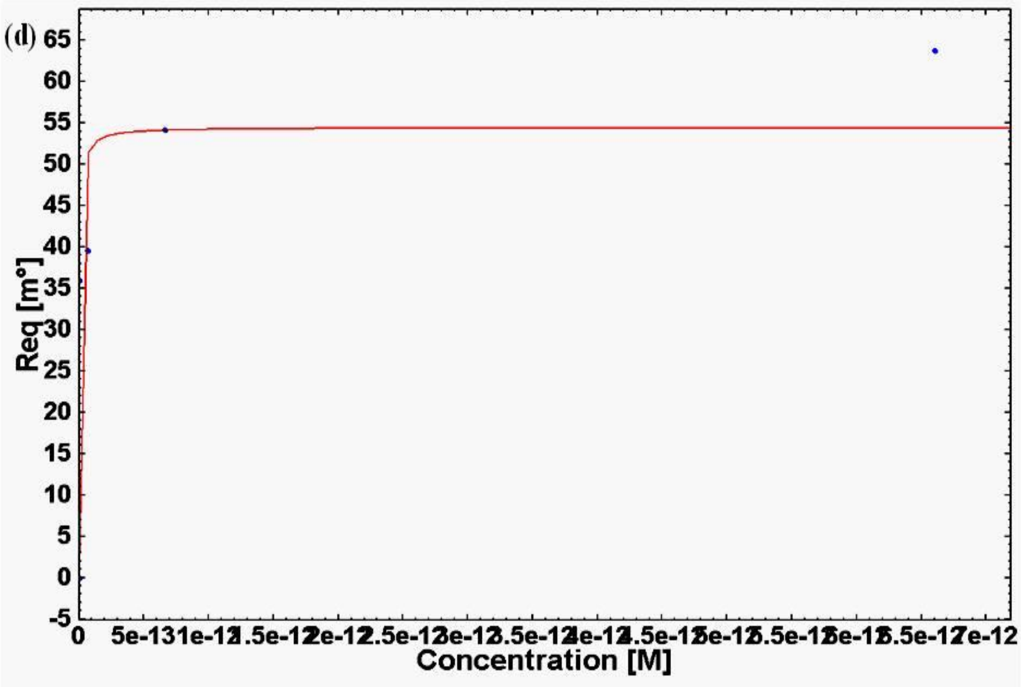

Supplementary Figure Captions

Supplementary Figure S1. Antibody titer estimation of pAbs developed against WC Ag of *Brucella* spp. **(a,b)** Titer against WC Ag of *Brucella abortus* S99 and, **(c,d)** against WC Ag of *Brucella melitensis* 16M in BALB/c mice and White New Zealand rabbit respectively. Two fold dilution of HIS resulted in polynomial regression co-efficient value, $R^2 = 1$ with linearity in variable trend between the OD values obtained as, $R^2 = 0.999$ at equation, $y = -0.009x^4 + 0.160x^3 – 0.878x^2 + 2.037x – 1.189$ and $R^2 = 0.994$ with $y = - 0.019x^3 + 0.233x^2 – 0.181x + 0.103$ for titer against WC Ag of *Brucella abortus* S99 and, $R^2 = 0.999$ at equation, $y = 0.012x^3 – 0.085x^2 + 0.252x – 0.130$ and $R^2 = 0.995$ with $y = - 0.005x^3 + 0.066x^2 – 0.071x + 0.266$ for titer against WC Ag of *Brucella melitensis* 16M in mice and rabbit respectively. The OD value of highest Ab titer obtained is 2 fold higher than the OD of blank (OD = 0.539 and 0.697 at ≥ 64,000 Ab titer against *Brucella abortus* S99 and, OD = 0.132 and 0.376 at ≥

64,000 Ab titer against *Brucella melitensis* 16M in BALB/c mice and White New Zealand rabbit respectively).

Supplementary Figure S2. SDS PAGE analysis of purified pAbs (developed against *Brucella* WC Ag), *Brucella* Sonicated (SA) and Cell Envelope (CE) Ag and Western blot characterisation of different *Brucella* antigens with mice IgG detection pAb. **(a,b)** Rabbit IgG pAb against WC Ag of *Brucella abortus* S99 and *Brucella melitensis* 16M (Lane 1 and 2) and mice IgG pAb against *Brucella abortus* S99 and *Brucella melitensis* 16M (Lane 3 and 4), Lane M with Fermentas #SM0671 protein marker. The Heavy and Light chain fragment of immunoglobulin at 50 and 25 kDa was obtained in purified IgG pAbs (IPA-TCA treated) along with protein albumin at 72 kDa as major serum protein in conventional SAS purified IgG pAbs generated against WC Ag of two *Brucella* spp. **(c,d)** SA and CE Ag of *Brucella melitensis* 16M (Lane 1 and 2) and *Brucella abortus* S99 (Lane 3 and 4) respectively, Lane M with Fermentas #SM0671 protein marker. The whole protein profile along with group 1, 2 and 3 major proteins was obtained [(88-94), (36-38), (31-34 and 25-27 kDa)] for both *Brucella* spp. **(e)** Western blot characterisation with mice IgG pAb for SA Ag of *Brucella abortus* S99 and *Brucella melitensis* 16M (Lane 1 and 2) along with immunoblot (Lane 1 and 2) respectively, Lane M with Fermentas #SM0671 protein marker and, **(f)** Western blot characterisation with mice IgG pAb for heat-boiled Ag of *Brucella melitensis* 16M (Lane 1 and 2) and *Brucella abortus* S99 (Lane 3 and 4) along with immunoblot of *Brucella melitensis* and *Brucella abortus* (Lane 1 and 2) respectively, Lane M with Fermentas #SM1811 protein marker. Both the immunoblots of sonicated and heat-boiled Ag of *Brucella* spp. are showing more prominently group 1 protein (88-94 kDa) of *Brucella*.

Supplementary Figure S3. Whole cell based S-ELISA assay. **(a)** For the detection of *Brucella abortus* S99 and, **(b)** For the detection of *Brucella melitensis* 16M WC Ag captured with species specific rabbit IgG pAb at 10 µg mL^{-1} concentration. Two fold serial diluted WC mice IgG pAb was used as detection Ab with initial 100 and 200 µg mL^{-1} of Ab concentration. The polynomial regression co-efficient value, $R^2 = 1$ at equation, $y = 0.033x^3 – 0.306x^2 + 1.045x – 0.615$ and, $R^2 =1$ with $y = 0.002x^5 – 0.060x^4 + 0.564x^3 – 2.494x^2 + 5.386x – 3.290$ for the detection of *Brucella abortus* and *Brucella melitensis* respectively, showing linearity between the values obtained during S-ELISA assay optimization. The highest OD value of >1 was obtained on WC detection of *Brucella melitensis* 16M as compared to OD value which is <1 for *Brucella abortus* S99 WC detection.

Supplementary Figure S4. Test validation of developed S-ELISA assay for *Brucella melitensis* 16M WC detection. **(a)** Validation with 10 fold serial diluted spiked WC Ag (10^8 CFU mL^{-1} to 10^1 CFU mL^{-1}) of *Brucella melitensis* 16M captured by rabbit IgG capture Ab at 10 µg mL^{-1} and, **(b)** rabbit IgG capture Ab at 20 µg mL^{-1} along with 50 µg mL^{-1} of mice IgG detection Ab to evaluate the effective concentration of capture pAb for *Brucella* WC detection in different clinical and non-clinical matrices. The polynomial regression co-efficient value, $R^2 = 0.953$ at equation, $y = - 0.002x^3 + 0.057x^2 – 0.500x + 2.026$ and $R^2 = 0.911$ with $y = - 0.007x^3 + 0.122x^2 – 0.652x + 1.438$ at 10 and 20 µg mL^{-1} concentration of rabbit IgG capture Ab respectively, used to determine the linear variable trend with comparatively more linear response at 10 µg mL^{-1} concentration at detection limit of 10^3 CFU mL^{-1} for *Brucella melitensis* 16M WC detection.

Supplementary Figure S5. Sensogram of SPR showing different steps for immobilization of mice IgG detection pAb. (**a,b)** Immobilization of mice IgG detection Ab developed against

WC Ag of *Brucella abortus* S99 and *Brucella melitensis* 16M (from left to right) respectively [Baseline, EDC-NHS activation, Washing, Antibody coupling, Washing, De-activation, Washing, Regeneration and Back to baseline] on 4-MBA modified SPR-Au chip surface. The resulted immobilization at 10 PPM concentration of mice IgG detection Ab is more parabolic for *Brucella melitensis* 16M WC pAb at a critical angle on SPR-Au chip surface.

Supplementary Figure S6. SPR sensor response and kinetic evaluation of immobilized *Brucella* CE Ag interaction with mice IgG detection pAb. **(a,b)** Interaction of immobilized *Brucella abortus* S99 and *Brucella melitensis* 16M CE Ag with different concentration of mice IgG detection Ab for both the *Brucella* spp. respectively at (a) 10^{-1} ng mL^{-1} (b) 1 ng mL^{-1} (c) 10^{-2} µg mL^{-1} (d) 10^{-1} µg mL^{-1} (e) 1 µg mL^{-1} in PBS. **(c,d)** SPR sensor response at equilibrium (at temperature - 25 °C and pH - 7.2**)** for immobilized *Brucella abortus* S99 and *Brucella melitensis* 16M CE Ag on interaction with different concentrations of mice IgG detection pAb of two *Brucella* spp. respectively for the evaluation of kinetic parameter.
